# Enhanced Replication of Mouse Adenovirus Type 1 following Virus-Induced Degradation of Protein Kinase R (PKR)

**DOI:** 10.1128/mBio.00668-19

**Published:** 2019-04-23

**Authors:** Danielle E. Goodman, Carla D. Pretto, Tomas A. Krepostman, Kelly E. Carnahan, Katherine R. Spindler

**Affiliations:** a Department of Microbiology and Immunology, University of Michigan, Ann Arbor, Michigan, USA; b Cellular and Molecular Biology Program, University of Michigan, Ann Arbor, Michigan, USA; Princeton University; Stony Brook University; University of Birmingham

**Keywords:** PKR, adenoviruses, eIF2alpha kinase, proteasome, protein degradation, protein stability

## Abstract

Protein kinase R (PKR) plays a major role in activating host immunity during infection by sensing double-stranded RNA (dsRNA) produced by viruses. Once activated by dsRNA, PKR phosphorylates the translation factor eukaryotic initiation factor 2α (eIF2α), halting cellular translation. Many viruses have methods of inhibiting PKR activation or its downstream effects, circumventing protein synthesis shutdown. These include sequestering dsRNA or producing proteins that bind to and inhibit PKR activation. Here we describe our finding that in multiple cell types, PKR was depleted during mouse adenovirus type 1 (MAV-1) infection. MAV-1 did not appear to be targeting PKR at the transcriptional or translational level, because total PKR mRNA levels and levels of PKR mRNA bound to polysomes were unchanged or increased during MAV-1 infection. However, inhibiting the proteasome reduced the PKR depletion seen in MAV-1-infected cells, whereas inhibiting the lysosome had no effect. This suggests that proteasomal degradation alone is responsible for PKR degradation during MAV-1 infection. Time course experiments indicated that the degradation occurs early after infection. Infecting cells with UV-inactivated virus prevented PKR degradation, whereas inhibiting viral DNA replication did not. Together, these results suggest that an early viral gene is responsible. Degradation of PKR is a rare mechanism to oppose PKR activity, and it has been described in only six RNA viruses. To our knowledge, this is the first example of a DNA virus counteracting PKR by degrading it.

## Abstract (precis)

The first line of defense in cells during viral infection is the innate immune system, which is activated by different viral products. PKR is a part of this innate immune system and is induced by interferon and activated by dsRNA produced by DNA and RNA viruses. PKR is such an important part of the antiviral response that many viral families have gene products to counteract its activation or the resulting effects of its activity. Although a few RNA viruses degrade PKR, this method of counteracting PKR has not been reported for any DNA viruses. MAV-1 does not encode virus-associated RNAs, a human adenoviral defense against PKR activation. Instead, MAV-1 degrades PKR, and it is the first DNA virus reported to do so. The innate immune evasion by PKR degradation is a previously unidentified way for a DNA virus to circumvent the host antiviral response.

## INTRODUCTION

Activation of protein kinase R (PKR) is a major innate immune response to viral infection. PKR is an interferon (IFN)-induced protein that is comprised of two major domains, namely, an N-terminal double-stranded RNA binding domain and a C-terminal serine/threonine kinase domain (1, 2). PKR binds to double-stranded RNA (dsRNA) (3–5), and, once bound, it becomes activated by dimerizing and autophosphorylating (6–9). When activated, PKR phosphorylates eukaryotic translation initiation factor 2α (eIF2α), causing inhibition of protein synthesis and reduced viral replication (10–13). Many viruses encode gene products that block PKR activation or inhibit its ability to phosphorylate eIF2α (14). A common mechanism is that of producing a viral protein that binds and sequesters dsRNA, blocking its interaction with PKR. Examples of this are vaccinia virus E3L (15–17), influenza virus NS1 (18, 19), and Ebola virus protein VP35 (20). Other viruses produce proteins or RNAs that bind directly to PKR to inhibit its activation, such as herpes simplex virus US11 (21, 22), HIV-1 Tat protein (23, 24) or *trans*-activation response element (TAR) RNA (25), and human adenovirus (hAd) virus-associated (VA) RNAs (10, 26–28).

Degradation of PKR by viruses is a less extensively documented method of regulating PKR. To date, PKR degradation has been reported in six RNA viruses: Toscana virus (TOSV) (29), Rift Valley fever virus (RVFV) (30–32), poliovirus (33, 34), foot-and-mouth disease virus (FMDV) (35, 36), encephalomyocarditis virus (EMCV [strain mengovirus]) (37, 38), and enterovirus 71 (39). RVFV and TOSV both degrade PKR via proteasomal mechanisms involving a viral nonstructural protein (NSs) (29, 32, 40). RVFV NSs recruits a SCF (SKP1-CUL1-F-box)^FBXW11^ E3 ubiquitin ligase to ubiquitinate PKR and target it to the proteasome, though PKR ubiquitination could not be demonstrated (32, 40). The mechanism for PKR proteasomal degradation by NSs has not been described for TOSV (29). FMDV uses the other major cellular protein degradation pathway, the lysosome, to degrade PKR during infection (36). Though the mechanism is unclear, expression of major FMDV protease 3C^pro^ is required for PKR degradation by the lysosome. However, 3C^pro^ does not interact with PKR, and its protease activity is not required for PKR degradation. Enterovirus A71 3C^pro^ causes PKR degradation by direct interaction (39). The mechanism of PKR depletion by poliovirus is unclear, though gene expression is required and the major poliovirus proteases (2A and 3C) are not directly involved (33). The mechanism by which mengovirus depletes PKR during infection is unknown (37, 38).

Adenoviruses are species specific, making the study of hAd pathogenesis difficult in an animal model. MAV-1 represents a useful alternative to study adenovirus pathogenesis (41–45). MAV-1 has molecular, genetic, and pathogenic similarities to and differences from hAd. Their genomic structures are similar at a gross level, and both contain early genes involved in pathogenesis and immune evasion. Pathogenically, their tropisms differ, with hAd infecting epithelial cells, leading to upper respiratory and GI tract infections and to conjunctivitis, while MAV-1 infects endothelial cells and monocytes, causing encephalitis and myocarditis. We and others have been investigating the adaptive and innate immune responses to MAV-1.

Human adenovirus VA RNAs bind PKR as a monomer, preventing its transautophosphorylation (46). However, MAV-1 does not produce VA RNAs (47), and whether MAV-1 induces PKR activation is not known. In our studies of MAV-1 pathogenesis and the innate response, we discovered that during MAV-1 infection, PKR was depleted from cells as early as 12 h postinfection (hpi). Total PKR mRNA levels and levels of PKR mRNA bound to polysomes were unchanged or increased during MAV-1 infection, suggesting that MAV-1 did not appear to be targeting PKR at a transcriptional or translational level. However, inhibiting the proteasome blocked the PKR depletion seen in MAV-1-infected cells, indicating that proteasomal degradation is responsible for PKR depletion during MAV-1 infection. We report results indicating that an early viral gene is likely responsible for mediating PKR degradation. To our knowledge, this is the first example of a DNA virus counteracting PKR by degrading it.

## RESULTS

### Viral DNA yield is increased in PKR^−/−^ mouse embryonic fibroblasts.

While PKR is an important part of the innate immune response, PKR^−/−^ cells in culture are not always more susceptible to viral infection than wild-type (WT) cells (48–50). PKR^−/−^ mouse embryonic fibroblasts (MEFs) show increased viral yields compared to wild-type MEFs when infected with vesicular stomatitis virus and influenza A virus (48, 49), but there is no change in viral yield during vaccinia virus infection compared to the results seen with wild-type cells (50). However, it was later discovered that the PKR^−/−^ MEF lines used were not complete PKR knockouts (51). There are two categories of PKR^−/−^ MEFs derived from knockout mice: N-PKR^−/−^ MEFs and C-PKR^−/−^ MEFs (51). The PKR^−/−^ MEFs derived from mice created in the Weissmann laboratory (52) are designated N-PKR^−/−^ MEFs, because the C-terminal fragment of PKR is still expressed and can be detected by immunoblotting when there is IFN induction (51). The fragment has the kinase catalytic activity of PKR, but it does not bind dsRNA (51). The PKR^−/−^ MEFs derived from mice created in the Bell laboratory (53) are designated C-PKR^−/−^ MEFs, because the N-terminal fragment of PKR is still expressed and can be detected by immunoblotting with specific PKR antibodies (51). The fragment is catalytically inactive, but it can still bind dsRNA (51). Susceptibility of these PKR^−/−^ MEFs to specific viruses may be dependent on the PKR mutation and the mechanism used by each virus to circumvent PKR.

**FIG 1:**
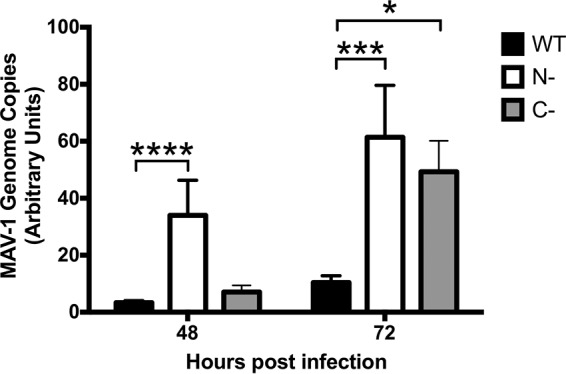
Viral DNA yield is increased in PKR^−/−^ mouse embryonic fibroblasts. PKR WT MEFs (WT), N-PKR^−/−^ MEFs (N-), and C-PKR^−/−^ MEFs (C-) were infected with MAV-1 at an MOI of 1 and collected at 48 and 72 hpi. DNA was purified from cell pellets and analyzed for MAV-1 genome copies by qPCR. The graph is representative of results from three experiments (14 biological replicates per cell line per time point). Error bars represent standard errors of the means (SEM). *, *P ≤ *0.05; ***, *P ≤ *0.0002; ****, *P ≤ *0.0001.

To determine whether PKR plays an important role during MAV-1 infection, we tested the susceptibility of both PKR^−/−^ MEF lines to MAV-1 infection. We infected wild-type MEFs, N-PKR^−/−^ MEFs, and C-PKR^−/−^ MEFs with MAV-1 at a multiplicity of infection (MOI) of 1 PFU/cell and collected cell pellets at 48 and 72 hpi. DNA was purified from the cell pellets and analyzed for MAV-1 genome copies by quantitative PCR (qPCR). N-PKR^−/−^ MEFs produced a significantly higher viral DNA yield than wild-type MEFs at 48 hpi, and both PKR mutant MEF lines had a significantly higher viral DNA yield than wild-type MEFs at 72 hpi (Fig. 1). Although we have not confirmed the production of truncated PKR proteins in the cells in our laboratory, the results shown in Fig. 1 indicate that PKR activation is an important antiviral response during MAV-1 infection *in vitro*.

### Mouse PKR is depleted during MAV-1 infection.

**FIG 2:**
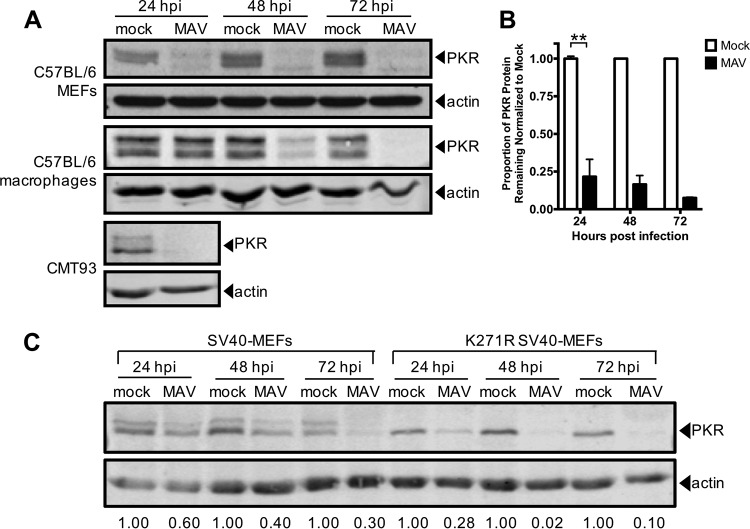
Mouse PKR is depleted during MAV-1 infection. (A) Cells (indicated at left) were infected with MAV-1 (MAV) at an MOI of 10 or were mock infected (mock). Cell lysates were collected at the indicated times and analyzed by immunoblotting with antibodies for PKR (B-10 for C57BL/6 MEFs and D-20 for C57BL/6 primary peritoneal macrophages and CMT93 cells) and actin. Blots are representative of results from a minimum of three independent experiments per cell line. (B) Densitometry quantitation of five of the C57BL/6 MEF immunoblots represented in panel A. Error bars represent SEM. *n* = 5 for 24 hpi, *n* = 4 for 48 hpi, and *n* = 2 for 72 hpi. **, *P ≤ *0.01. (C) Untreated SV40 MEFs or kinase-dead (K271R) SV40 MEFs were infected with MAV-1 at an MOI of 10. Cell lysates were immunoblotted as described for panel A with PKR D-20. The numbers below the blots represent the proportion of PKR protein for each time point, normalized to actin, and the mock PKR protein levels from the corresponding time point.

To determine whether MAV-1 affects PKR during infection, we infected several cell types and analyzed PKR protein expression. We infected immortalized C57BL/6 MEFs, C57BL/6 primary peritoneal macrophages, and CMT93 cells (mouse rectal carcinoma cells) with MAV-1 at an MOI of 10 and collected cell lysates 24, 48, and 72 hpi. We analyzed cell lysates for the presence of PKR by immunoblotting using a polyclonal antibody that detects mouse PKR. We probed blots with antibodies to actin as a loading control. To our surprise, in C57BL/6 MEFs, PKR was almost completely depleted from lysates at 24 hpi and remained depleted through 72 hpi (Fig. 2A and B). PKR was also depleted compared to the levels seen with mock infection at 24 and 48 hpi in C57BL/6 MEFs infected at MOIs of 2 and 5 (see Fig. S1 in the supplemental material). We also observed depletion of PKR in other cell types. In CMT93 cells, PKR was nearly undetectable at 24 hpi (Fig. 2A). PKR levels were decreased in C57BL/6 primary peritoneal macrophages at 48 hpi compared to mock-infected lysates, and PKR was absent in infected lysates at 72 hpi (Fig. 2A). This indicates that MAV-1 causes PKR depletion during infection.

10.1128/mBio.00668-19.1 FIG S1 PKR is depleted at MOIs of 2, 5, and 10. C57BL/6 MEFs were infected with MAV-1 at an MOI of 2, 5, or 10 or were subjected to mock infection (mock). Cell lysates were analyzed by immunoblotting with antibodies for PKR (B-10) and actin. Data from densitometry quantitations performed using ImageJ are listed below each lane; for each time point, the infected samples were normalized to the mock-infected samples. The PKR blot image was uniformly adjusted to a brightness of 150 in Adobe Photoshop. Download FIG S1, PDF file, 2.2 MB. Copyright © 2019 Goodman et al. 2019 Goodman et al. This content is distributed under the terms of the Creative Commons Attribution 4.0 International license.

To determine whether the kinase activity of PKR is important for the depletion, we assayed infection of MEFs expressing a mutant form of mouse PKR with a point mutation in the kinase domain (K271R) (54). These cells, designated K271R simian virus 40 (SV40) MEFs, showed an even higher rate of depletion of PKR than the WT SV40 MEFs (Fig. 2C). At 24 hpi, 28% of PKR remained in K271R SV40 MEFs compared to 60% in the WT SV40 MEFs. The fraction remaining at 72 hpi in K271R SV40 MEFs was 10% compared to 30% in WT SV40 MEFs (Fig. 2C). This indicates that the PKR kinase does not have to be functional to be depleted during MAV-1 infection. Also, comparing the PKR immunoblot bands in the mock-infected WT SV40 MEFs and mutant K271R SV40 MEFs suggests that the upper band of the PKR doublet usually seen in wild-type cells is a phospho-PKR band, because only the lower band of the PKR doublet is seen in kinase-dead mutant K271R SV40 MEFs. The data in Fig. 2A and C thus indicate that both PKR and phospho-PKR are depleted during MAV-1 infection.

### MAV-1 does not cause PKR depletion by reducing steady-state levels of PKR mRNA.

**FIG 3:**
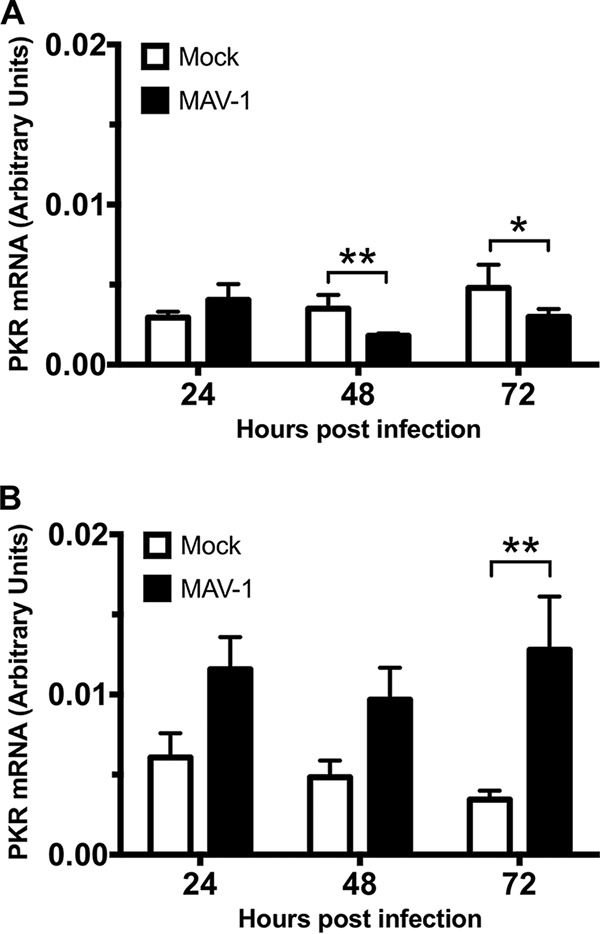
MAV-1 does not cause PKR depletion by reducing steady-state levels of PKR mRNA at times when the protein levels are already reduced. MEFs (A) or isolated primary peritoneal macrophages (B) were harvested and infected with MAV-1 at an MOI of 10 or mock infected. The cell pellets were collected, and RNA was isolated. cDNA was generated from the RNA, and qPCR was used to quantitate PKR mRNA levels. Each graph represents 5 to 7 replicates for each time point from three pooled experiments. Error bars show the SEM. *, *P ≤ *0.05; **, *P ≤ *0.01.

To determine the mechanism of PKR depletion, we first assayed whether the reduction in the level of PKR protein during MAV-1 infection was due to reduced PKR mRNA steady-state levels. We mock-infected or infected C57BL/6 MEFs and primary peritoneal macrophages at an MOI of 10 and collected cell lysates at 24, 48, and 72 hpi. We synthesized cDNA from RNA purified from these cell lysates and assayed for PKR mRNA by qPCR. The PKR mRNA levels in C57BL/6 MEFs were similar between mock-infected and infected lysates at 24 hpi (Fig. 3A), a time point at which the PKR protein levels were already greatly reduced in the infected lysates compared to mock-infected lysates (Fig. 2). Although the PKR mRNA levels were depleted 33% at 48 hpi and 40% at 72 hpi in MAV-1-infected lysates compared to mock-infected lysates, this does not correlate to the 84% and 94% reductions, respectively, in PKR protein levels seen at those time points (Fig. 2B). PKR mRNA levels in C57BL/6 primary peritoneal macrophages in the infected lysates were 2 to 3 times higher than the levels in the mock-infected lysates at all three time points assayed (Fig. 3B), even though the PKR protein was almost completely depleted in infected lysates at 72 hpi (Fig. 2). This represents evidence that MAV-1 was not causing PKR protein depletion by reducing PKR steady-state mRNA levels during infection.

### MAV-1 infection effects on PKR translation.

**FIG 4:**
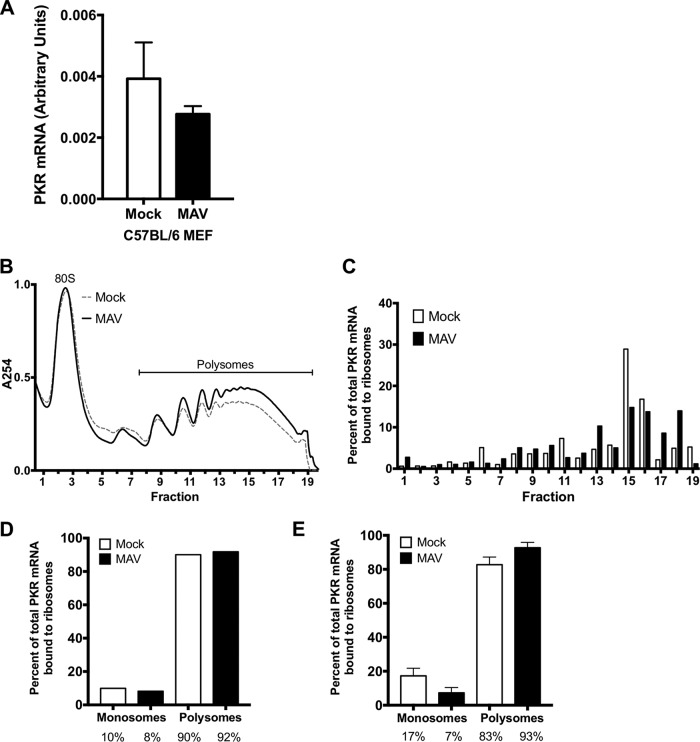
MAV-1 infection does not affect PKR translation. (A) C57BL/6 MEFs were infected with MAV-1 (MAV) at an MOI of 5 or were mock infected (mock) and collected at 48 hpi. Cells were lysed, and cleared lysates from three 10-cm-diameter plates were layered onto 25% sucrose and centrifuged to pellet ribosomes. RNA was purified from the pellets, cDNA was generated from the RNA, and qPCR was used to quantitate the PKR mRNA levels. The graph represents 9 replicates for each time point, pooled from 3 independent experiments. Error bars show the SEM. (B) C57BL/6 MEFs were infected with MAV-1 (MAV) at an MOI of 2 or were mock infected (mock). Cells were collected at 25 hpi and lysed; cleared lysates were layered onto 10%-to-50% sucrose gradients and centrifuged. Gradients were collected from the top and pumped through a UV spectrophotometer, and 34 fractions were collected. The gradients are displayed with the bottom fractions indicated to the right. The UV trace of the first 10 fractions (including 40S and 60S ribosomal subunits) is not shown. (C) RNA was purified from each fraction of the gradients represented in panel B. cDNA was generated from the RNA, qPCR was used to quantitate PKR mRNA in each fraction, and the results are displayed as the percentages of total PKR mRNA associated with ribosomes. Panels B and C present results from one experiment representative of 3 independent experiments. (D) Percentage of total PKR mRNA associated with monosomes (fractions 1 to 6) and polysomes (fractions 7 to 19) from the trial represented in panels B and C (pooled for mock-infected and infected samples). The percentages represented by each bar are displayed below each bar. (E) Pooled monosome and polysome data (as described for panel D) from three independent experiments. Error bars show the SEM. The percentages represented by each bar are displayed below each bar. There were no significant differences between the mock-infected samples and the infected samples (A and E).

Because MAV-1 did not cause reductions in the PKR mRNA steady-state levels, we determined whether MAV-1 causes PKR depletion by reducing translation of its mRNA. We first assayed total PKR mRNA bound to ribosomes during infection. C57BL/6 MEFs were mock infected or infected at an MOI of 5, and lysates were collected at 48 hpi in the presence of cycloheximide to keep the mRNA bound to the ribosomes (55). Lysates were centrifuged through 25% sucrose to pellet ribosomes, and RNA was purified from the pellets. The purified RNAs were used to generate cDNA, which we assayed for PKR mRNA by qPCR. As a control for pelleting of ribosomes, we assayed the pellets and sucrose cushion supernatants by immunoblotting with antibodies to ribosomal protein RPL7. We confirmed that RPL7 was present only in the pellets and not in the supernatants (Fig. S2). There was no significant difference between the amount of PKR mRNA in the ribosome pellet of mock-infected lysates and the amount in that of MAV-1-infected lysates (Fig. 4A).

10.1128/mBio.00668-19.2 FIG S2 Ribosome pelleting. To confirm that most ribosomes ended up in the pellet after centrifugation through sucrose (Fig. 4A), a sample of the pellet and a sample of the corresponding supernatant were analyzed by immunoblotting with antibodies for RPL7 (ribosomal protein L7; Abcam catalog no. ab72550) (1:2,000). Download FIG S2, PDF file, 3.2 MB. Copyright © 2019 Goodman et al. 2019 Goodman et al. This content is distributed under the terms of the Creative Commons Attribution 4.0 International license.

To confirm the results seen in total mRNA bound to ribosomes, we also centrifuged cell extracts on sucrose gradients to generate polysome profiles. This enabled us to analyze levels of PKR mRNA associated with actively translating ribosomes during infection. C57BL/6 MEFs were mock infected or infected at an MOI of 2, and lysates were collected at 24 hpi in the presence of cycloheximide, as described above. The levels of RNA content for mock-infected and infected lysates were estimated by NanoDrop spectrophotometry, and equivalent optical density (OD) amounts of RNA were centrifuged on 10% to 50% sucrose gradients to sediment 40S and 60S ribosomal subunits, 80S ribosomes (monosomes), and polyribosomes (polysomes). A typical polysome profile was obtained (Fig. 4B). RNA was purified from fractions containing monosomes and polysomes and then used to generate cDNA, which we assayed for PKR mRNA by qPCR (Fig. 4C). As a control, GAPDH mRNA was measured by qPCR, and PKR mRNA levels in each fraction were normalized to the GAPDH mRNA content. When the data representing the percentages of PKR mRNA bound to ribosomes were pooled into monosome and polysome fractions and analyzed (Fig. 4D), 90.1% and 91.8% were bound to polysomes (fractions 7 to 19) for mock-infected and infected samples, respectively, compared to 9.9% and 8.2% bound to monosomes (fractions 1 to 6). We performed two additional polysome gradient analyses. The pooled data from all three analyses (Fig. 4E) were similar to the data shown in Fig. 4D; i.e., 82.7% and 92.7% of PKR mRNA were bound to mock-infected and infected polysomes, respectively, compared to 17.3% and 7.3% bound to monosomes. Thus, PKR protein depletion during MAV-1 infection does not appear to stem from a decrease in PKR mRNA translation.

We also assayed whether PKR mRNA might have a signal that would reduce its translation during MAV-1 infection. We constructed a plasmid that positioned sequence corresponding to the 5′ untranslated region (UTR) of PKR mRNA upstream of a reporter nanoluciferase gene (56), transfected it into C57BL/6 MEFs, and then infected with MAV-1. Compared to cells transfected with a control plasmid with the human β-globin 5′ UTR upstream of the reporter nanoluciferase, there was no significant difference in luciferase activity between mock-infected and infected samples (Fig. S3). These data suggest that MAV-1 was not affecting PKR translation through interaction with the 5′ UTR of PKR. The data are consistent with the ribosome pellet and polysome data indicating that MAV-1 infection does not reduce PKR mRNA translation.

10.1128/mBio.00668-19.3 FIG S3 PKR mRNA 5′ UTR presence does not result in altered reporter protein levels upon MAV-1 infection. (A) C57BL/6 MEFs or (B) CMT93 cells were cotransfected with pmPKR5UTRfullNL or AUG-NL-3xFLAG and pGL4.13 using jetPRIME reagents (Polyplus catalog no. 114-15) and the standard Polyplus protocol, with 200 ng total of plasmid and 300 µl of jetPRIME reagent per 35-mm-diameter well. At 24 h after transfection, the cells were infected with MAV-1 at an MOI of 10. At 24 hpi, cells were lysed in Glo lysis buffer (Promega Corp.) (70 µl/well). After lysing, 25 µl of each lysed sample and 25 µl of OneGlo or NanoGlo (Promega Corp.) were added to two wells in a black 96-well plate (Fisher Scientific catalog no. 07-000-634). After 5 min, the plate was read on a Promega GloMax luminometer. Levels of relative light units from the pmPKR5UTRfullNL plasmid were normalized to the firefly luciferase and positive-control plasmids. Graphs are representative of 7 to 9 biological replicates per treatment group. Download FIG S3, PDF file, 0.1 MB. Copyright © 2019 Goodman et al. 2019 Goodman et al. This content is distributed under the terms of the Creative Commons Attribution 4.0 International license.

### PKR is depleted by proteasomal degradation during MAV-1 infection.

**FIG 5:**
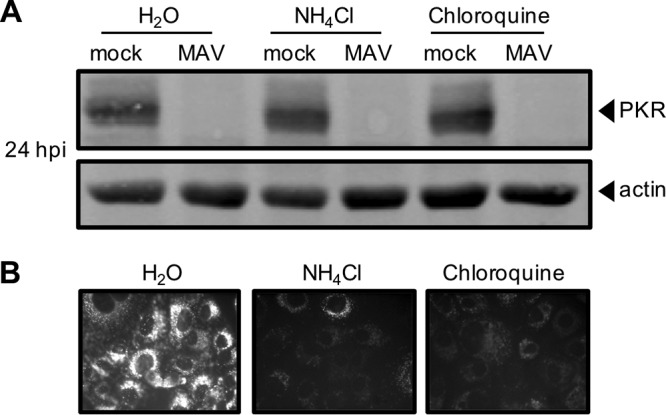
PKR is not depleted by lysosomal degradation during MAV-1 infection. (A) CMT93 cells were infected with MAV-1 (MAV) at an MOI of 10 or were mock infected (mock) and treated with 10 mM ammonium chloride or 60 µM chloroquine to inhibit lysosomal degradation (or with water as a control). Cell lysates were analyzed by immunoblotting with antibodies for PKR (D-20) and actin. Blots are representative of results from three independent experiments. (B) Inhibitors were tested for activity using a DQ BSA assay; the DQ BSA molecule fluoresces only if lysosomal degradation is functional. Uninfected cells were treated as indicated and imaged by fluorescence microscopy.

There are two main proteolysis pathways in cells: proteasomal degradation and lysosomal degradation (57). To determine whether MAV-1 depletes PKR by either protein degradation pathway, we first assayed whether PKR is lysosomally degraded as follows. CMT93 cells were mock infected or infected with MAV-1 and treated at the time of infection with a lysosome inhibitor (ammonium chloride or chloroquine) or water (as a control). At 24 hpi, we collected lysates and analyzed them by immunoblotting with antibodies to PKR. In the presence of the lysosomal degradation inhibitors, PKR was depleted by 24 hpi (Fig. 5A), indicating that lysosomal degradation was not the cause of PKR depletion during MAV-1 infection. We confirmed that the inhibitor treatment did block lysosomal degradation by incubating cells with dye-quenched bovine serum albumin (DQ BSA) in addition to the lysosomal inhibitors. DQ BSA is self-quenched until it is digested in the lysosome (58, 59), and imaging confirmed that the cells treated with lysosome inhibitors did not fluoresce but that the cells treated with the vehicle control (H_2_O) did, as expected (Fig. 5B).

**FIG 6:**
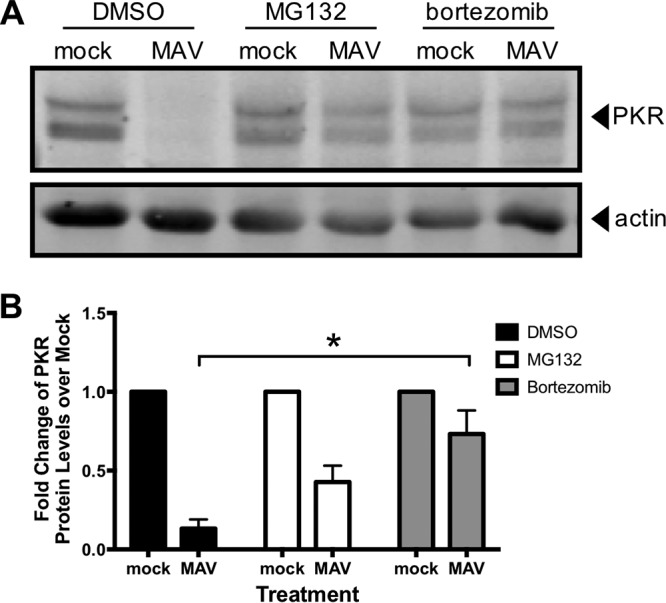
PKR is depleted by proteasomal degradation during MAV-1 infection. (A) C57BL/6 MEFs were infected with MAV-1 (MAV) at an MOI of 10 or were mock infected (mock) and treated with DMSO (vehicle for inhibitors), 1 µM MG132, or 1 µM bortezomib. Cell lysates were then analyzed by immunoblotting with antibodies for PKR (D-20) and actin. Blots are representative of results from four independent experiments. (B) Densitometry quantitation of four independent experiments. Treatment with bortezomib significantly inhibited PKR depletion in MAV-1-infected cells. *, *P ≤ *0.05.

Next, we examined whether proteasomal degradation is responsible for the degradation of PKR by using proteasome inhibitors MG132 and bortezomib. These inhibit proteasome activity by binding to the active sites in the 20S subunit and blocking the proteolytic activity (60–62). We mock infected or infected C57BL/6 MEFs with MAV-1 and treated with MG132 or bortezomib and dimethyl sulfoxide (DMSO) at the time of infection. At 24 hpi, we collected lysates and analyzed them by immunoblotting for PKR protein levels. While PKR was depleted in the control DMSO-treated MAV-1-infected cells as expected, PKR protein was present in the MG132- and bortezomib-treated cells at levels comparable to those in the mock-infected cells (Fig. 6). To rule out the possibility that PKR was present (not depleted) because the virus infection itself was inhibited by MG132 or bortezomib, we assayed viral replication of MAV-1 with MG132 and bortezomib treatment by qPCR of viral DNA. Viral replication levels were equivalent in all three treatment groups (Fig. S4), indicating that the treatments did not affect the ability of the virus to productively infect the cells. Taken together, these data indicate that MAV-1 infection results in PKR depletion by causing PKR to be degraded by the proteasome during infection.

10.1128/mBio.00668-19.4 FIG S4 Treatment with MG132 and bortezomib does not affect MAV-1 replication at 24 hpi. C57BL/6 MEFs were infected with MAV-1 at an MOI of 10 and treated with DMSO (vehicle for inhibitors) or with 1 µM MG132 or bortezomib and were collected at 24 hpi. DNA was purified from cell pellets and analyzed for MAV-1 genome copies by qPCR. The graph is representative of results from five biological replicates per treatment group. Error bars represent standard errors of the means (SEM). *, *P ≤ *0.05. Download FIG S4, PDF file, 0.1 MB. Copyright © 2019 Goodman et al. 2019 Goodman et al. This content is distributed under the terms of the Creative Commons Attribution 4.0 International license.

A signal for proteasomal degradation is the conjugation of ubiquitin to a protein (63, 64). We examined by immunoblotting whether PKR is ubiquitinated. We detected ubiquitination of a positive control, mouse p53, which is degraded in the presence of MAV-1 proteins (65). However, even with the use of epitope-tagged ubiquitin (66) and MG132 treatment, we were unable to detect PKR ubiquitination during infection (Fig. S5). This is consistent with an inability to detect PKR ubiquitination when it is degraded during RVFV infection (32). Although RVFV NSs is known to recruit an E3 ligase to PKR, the authors of that study reported that ubiquitinated PKR is undetectable. Therefore, the cellular degradation signal for PKR remains unclear.

10.1128/mBio.00668-19.5 FIG S5 PKR is not detectably ubiquitinated during MAV-1 infection. CMT93 cells were transfected with a GFP-ubiquitin plasmid using standard Polyplus transfection protocols. At 24 h posttransfection (hpt), the cells were infected with MAV-1 (MAV) at an MOI of 5 or were mock infected (mock) and treated with 10 µM MG132 at 6 hpi. Cell lysates were collected at 12 hpi and immunoprecipitated with (A) PKR (D-20) or an isotype control antibody or (B) p53 (DO-1) or an isotype control antibody. Immunoprecipitated samples were analyzed by immunoblotting with GFP, ubiquitin, isotype, PKR (B-10), or p53 (1801) antibodies. The input lane (mock input) contained a 0.008 volume of mock-infected lysate (relative to the volume in the immunoprecipitations). For panel B, GFP and p53 were probed on separate duplicate blots. Download FIG S5, PDF file, 6.8 MB. Copyright © 2019 Goodman et al. 2019 Goodman et al. This content is distributed under the terms of the Creative Commons Attribution 4.0 International license.

### PKR is actively depleted early in infection.

**FIG 7:**
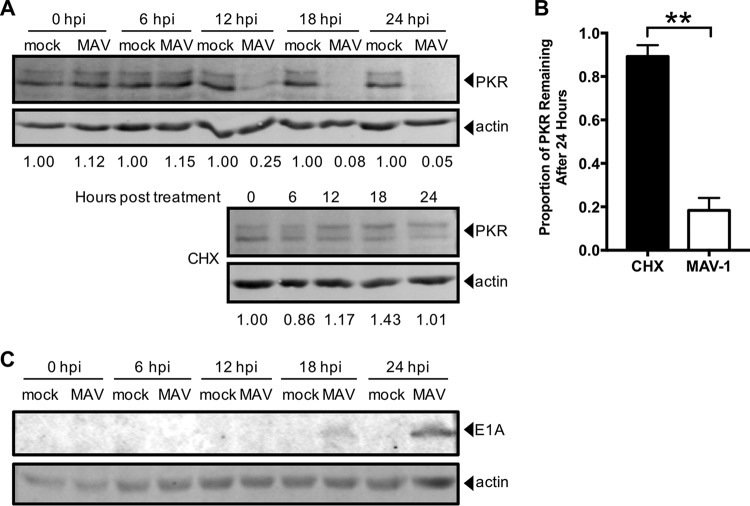
PKR is actively depleted early in infection. (A) CMT93 cells were infected with MAV-1 (MAV) at an MOI of 10 or were mock infected (mock) (top), or uninfected cells were treated with 50 µg/ml cycloheximide (CHX, bottom) to inhibit elongation of protein synthesis. Cell lysates were analyzed by immunoblotting with antibodies for PKR (D-20) and actin. Blots are representative of results from five independent experiments. (B) Densitometry quantitation of five independent experiments; **, *P ≤ *0.01. (C) CMT93 cell lysates from the experiment performed as described in the panel A legend were analyzed with a second immunoblotting step with antibodies for E1A and actin. Blots are representative of results from four replicates from two independent experiments. The E1A blot image was uniformly adjusted to a brightness value of 30 and a contrast value of 5 in Adobe Photoshop.

We investigated the time point at which proteasomal degradation of PKR occurs. Early viral proteins are expressed prior to viral DNA replication, which is then followed by late viral protein expression. First, we examined the kinetics of PKR degradation to determine whether an early or late viral protein was likely responsible. We mock infected and infected CMT93 cells with MAV-1 at an MOI of 10, collected lysates every 6 h for 24 h, and analyzed them by immunoblotting with antibodies to PKR or MAV-1 early region 1A (E1A) protein, the first viral protein made during infection (67). PKR degradation in the infected cells was first detected at 12 hpi (Fig. 7A), and quantitation of the results of five independent experiments showed that ∼20% of the starting levels of PKR protein remained at 24 hpi (Fig. 7B).

In parallel, to determine the half-life of PKR in uninfected CMT93 cells, we treated CMT93 cells with cycloheximide to halt protein translation and thus production of new PKR. We collected lysates every 6 h for 24 h and analyzed by immunoblotting with antibodies to PKR. After 24 h of cycloheximide treatment, approximately 90% of the starting levels of PKR protein remained (Fig. 7A, bottom, and 7B). Comparing the results from MAV-1 infection (Fig. 7A, top, and 7B) and cycloheximide treatment of uninfected cells (Fig. 7A, bottom, and 7B), we conclude that MAV-1 was actively depleting PKR protein early in infection. E1A was detected by immunoblotting at 18 hpi (Fig. 7C), whereas viral DNA replication was first detected at 24 hpi in CMT93 cells (Fig. S6). Thus, the 18-hpi time point is considered an early time point during MAV-1 infection of CMT93 cells, prior to DNA replication, suggesting the involvement of an early viral protein in PKR depletion.

10.1128/mBio.00668-19.6 FIG S6 Viral DNA replication can be detected at 24 hpi by qPCR. CMT93 cells were infected with MAV-1 (MAV) at an MOI of 10 and collected every 6 h for 24 h. DNA was purified from cell pellets and analyzed for MAV-1 genome copies by qPCR. The graph is representative of results from four to five biological replicates per treatment group. Error bars represent standard errors of the means (SEM). Download FIG S6, PDF file, 0.1 MB. Copyright © 2019 Goodman et al. 2019 Goodman et al. This content is distributed under the terms of the Creative Commons Attribution 4.0 International license.

### An early viral function is required for PKR depletion by MAV-1.

**FIG 8:**
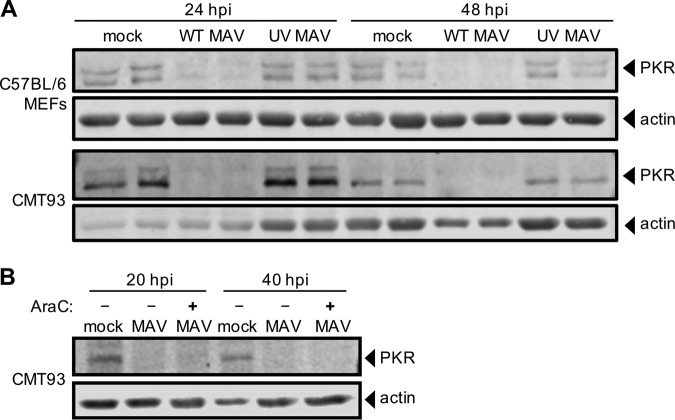
Early gene expression is required for PKR depletion by MAV-1. (A) Cells (as indicated at left) were infected with WT MAV-1 (WT MAV) or UV-inactivated MAV-1 (UV MAV) at an MOI of 10 or were mock infected (mock). Cell lysates were analyzed by immunoblotting with antibodies for PKR (D-20) and actin. Two independent wells were infected for each condition at both time points. (B) CMT93 cells were infected with WT MAV-1 (MAV) at an MOI of 10 or were mock infected (mock). Infected cells were also treated (+) or not (-) with 20 µg/ml cytosine arabinoside (AraC), an inhibitor of DNA synthesis. Cell lysates were analyzed with antibodies for PKR and actin.

To determine whether viral gene expression or DNA replication is required for PKR degradation during infection, we infected C57BL/6 MEFs and CMT93 cells with UV-inactivated MAV-1 (which does not replicate viral DNA; Fig. S7). We infected cells at an MOI of 10 with WT MAV-1 or UV-inactivated MAV-1 and analyzed lysates from 24 and 48 hpi by immunoblotting for PKR protein levels. In both cell types, while PKR was degraded by 24 hpi in the cells infected with WT MAV-1, PKR protein levels were unaffected at both time points in cells infected with UV-inactivated MAV-1 (Fig. 8A). This suggested that either gene expression or DNA replication was required for PKR degradation during MAV-1 infection.

10.1128/mBio.00668-19.7 FIG S7 UV-inactivated virus does not replicate viral DNA. (A) C57BL/6 MEFs or (B) CMT93 cells were infected with WT MAV-1 (WT) or UV-inactivated MAV-1 (UV) at an MOI of 10 and collected at the indicated times. DNA was purified from cell pellets and analyzed for MAV-1 genome copies by qPCR. Graphs are representative of results from three to four biological replicates per treatment group. Error bars represent standard errors of the means (SEM). Download FIG S7, PDF file, 0.1 MB. Copyright © 2019 Goodman et al. 2019 Goodman et al. This content is distributed under the terms of the Creative Commons Attribution 4.0 International license.

We addressed whether viral DNA replication is needed for PKR degradation. We mock infected or infected CMT93 cells with MAV-1 at an MOI of 10 and treated them with cytosine arabinoside (araC) at the time of infection to inhibit DNA synthesis (68, 69). This would allow the virus to infect the cell and produce early viral proteins, but would inhibit viral DNA replication and prevent late protein synthesis. We collected lysates at 20 and 40 hpi and analyzed them by immunoblotting. We confirmed that araC treatment resulted in no late protein synthesis by immunoblotting for late virion proteins (Fig. S8). In samples treated with araC, PKR degradation was seen at 20 and 40 hpi (Fig. 8B), indicating that DNA replication was not required for PKR degradation. Taken together, the results shown in Fig. 8 are consistent with early viral gene expression prior to DNA replication being involved in induction of PKR degradation by MAV-1.

10.1128/mBio.00668-19.8 FIG S8 AraC treatment inhibited late protein expression. CMT93 cells were infected with WT MAV-1 (MAV) at an MOI of 10 or were mock infected (mock). Infected cells were also treated (+) or not (-) with 20 µg/ml cytosine arabinoside (araC), an inhibitor of DNA synthesis. Cell lysates were analyzed with antibodies for late virion proteins (AKO1-103; 1:1,000). Arrowheads indicate late viral proteins. Download FIG S8, PDF file, 2.3 MB. Copyright © 2019 Goodman et al. 2019 Goodman et al. This content is distributed under the terms of the Creative Commons Attribution 4.0 International license.

## DISCUSSION

We have demonstrated here that PKR is antiviral in MAV-1 infections of cultured cells. Surprisingly, MAV-1 infection of primary and established cultured cells depleted PKR. The depletion was not due to reduced steady-state levels or reduced translation of PKR mRNA. Instead, we showed that PKR depletion is inhibited by proteasome inhibitors, implicating proteasomal degradation of PKR. Several lines of evidence suggest that the degradation is due to a viral early function.

PKR is an IFN-inducible gene product that is an important component of the innate immune response (1, 48). However, not all viruses, including EMCV and vaccinia virus, have increased virulence in PKR^−/−^ MEFs (50, 70). While hAds produce VA RNAs that inhibit PKR antiviral activity during infection (10, 71), MAV-1 does not produce such VA RNAs, and how MAV-1 infection is affected by PKR is first described in this report. When we infected PKR^−/−^ MEFs with MAV-1, viral DNA yields were 5 to 6 times higher than the viral DNA yields from wild-type MEFs (Fig. 1), indicating that PKR plays an antiviral role during MAV-1 infection. The viral DNA yield from the N-PKR^−/−^ MEFs was nearly 4 times higher than the yield from the C-PKR^−/−^ MEFs at 48 hpi, but by 72 hpi the viral DNA yields from the two types of PKR^−/−^ MEFs were similar to each other and were significantly increased compared to the yield seen with wild-type MEFs (Fig. 1). This difference in viral replication kinetics between the two types of PKR^−/−^ MEFs may be due to differences in the expression and activity levels of the PKR fragments reportedly produced by them; we have not assayed PKR fragment production in our cells.

We examined PKR protein levels during MAV-1 infection and found that PKR was depleted from the cells as early as 12 hpi (Fig. 7). Depletion was seen in a wide variety of cell types, including immortalized C57BL/6 MEFs, primary C57BL/6 peritoneal macrophages, and CMT93 mouse colon carcinoma cells. Once depleted, the PKR protein levels never returned to the levels measured for the mock-infected cells during infection. Activation (transautophosphorylation) of PKR (6–9) was not required for this depletion, because kinase-dead mouse PKR was also depleted from K271R SV40 MEFs during infection (Fig. 2C). Both PKR and phospho-PKR were depleted in all cell types examined.

We examined several possible explanations for the depletion of PKR protein, including PKR mRNA levels and alterations in translation. PKR mRNA levels remained unchanged during MAV-1 infection in C57BL/6 MEFs at 24 hpi and were increased in primary C57BL/6 peritoneal macrophages during MAV-1 infection (Fig. 3), corresponding to the times when the PKR protein levels were depleted (Fig. 2A). While the PKR mRNAs in C57BL/6 MEFs were depleted 33% at 48 hpi and 40% at 72 hpi compared to mock-infected lysates, this was not sufficient to explain the 84% and 94% reductions, respectively, in PKR protein levels at those time points (Fig. 2B). More likely, the reduction in PKR steady-state mRNA levels at the late infection time points can be attributed to other effects from viral infection, including the degradation or inhibition of proteins that induce PKR expression. For example, p53 is capable of binding to the PKR promoter and inducing its expression (72), but MAV-1 proteins cause p53 proteolysis (65). Together, our results from C57BL/6 MEFs and macrophages suggest that the virus does not cause PKR protein depletion by reducing PKR steady-state mRNA levels.

The differences in total PKR mRNA levels between C57BL/6 MEFs and primary peritoneal macrophages during infection (Fig. 3) were possibly due to the fact that macrophages are immune cells whereas MEFs are not. PKR protein took almost 3 times as long to be completely degraded during infection in macrophages than in MEFs (72 h versus 24 h) (Fig. 2A). Total PKR mRNA levels were 2 to 3 times higher in MAV-1-infected macrophages than in mock-infected macrophages, unlike the MEFs, where total PKR mRNA levels were unchanged or were reduced 33% to 40% during MAV-1-infection compared to the mock-infected MEFs. Since PKR is an IFN-stimulated gene (1, 2), the higher levels of total PKR mRNA seen during infection in the macrophages suggest IFN induction. This suggests that the immune response mounted by the macrophages was greater than the immune response in the MEFs and could help explain why PKR took longer to degrade in macrophages than in MEFs.

We considered whether the reduced PKR levels were due to reduced PKR protein translation. There was no change in the total amount of PKR mRNA bound to ribosomes during infection compared to uninfected cells, and there was also no significant change in the amount of actively translating PKR mRNA during infection (Fig. 4). We also found that the 5′ UTR of mouse PKR placed upstream of a reporter gene produced the same amounts of reporter with and without MAV-1 infection. These data indicate that there are not translational effects of MAV-1 infection on PKR protein levels that could explain the depleted PKR levels that we observed.

Inhibiting lysosomal degradation resulted in no change in PKR depletion in infected cells (Fig. 5A), but adding proteasome inhibitors preserved PKR protein within cells (Fig. 6A and B). This indicates that PKR is degraded not by lysosomal degradation during viral infection but by proteasomal degradation. Though PKR degradation was due to proteasome activity during MAV-1 infection, we were unable to demonstrate PKR ubiquitination, although we did detect ubiquitination of mouse p53 (see Fig. S5 in the supplemental material). This inability to demonstrate PKR ubiquitination could be explained if at any given moment there were only low levels of ubiquitinated PKR present in the cell. Perhaps increasing the time spent under conditions of MG132 treatment could increase the amounts of ubiquitinated proteins enough that PKR ubiquitination could be seen. However, our inability to detect ubiquitinated PKR is consistent with a similar inability to identify PKR ubiquitination by RVFV NSs, even though NSs is known to recruit an E3 ligase to PKR (32). Alternatively, it is possible that in MAV-1 infection, PKR is degraded in a ubiquitin-independent manner, possibly because of the presence of intrinsic disordered regions of PKR or binding of regulating proteins to PKR that target proteins to the proteasome (73, 74).

Our experiments indicated that MAV-1 actively depletes PKR early in infection. Ongoing experiments are focused on determining the MAV-1 early protein(s) responsible for PKR degradation. Two possibilities are represented by E4 proteins, the homologs of hAd E4orf6 and E4orf3, which we originally termed E4orfa/b and E4orfa/c, respectively (75). In human adenovirus, E4orf6 interacts with another early hAd protein, E1B 55K, to participate in an E3 ligase complex that ubiquitinates and degrades p53 via proteasomal degradation (76, 77). When MAV-1 E4orf6, E1B 55K, and mouse p53 are introduced by transfection into human cells, all three proteins interact and mouse p53 is degraded (65). If MAV-1 E4orf6 and E1B 55K form a similar complex in mouse cells, it may also degrade PKR. We have preliminary evidence indicating that mouse p53 is ubiquitinated in C57BL/6 MEFs during MAV-1 infection, which suggests that the mouse p53 degradation seen in human cells could be paralleled by degradation of endogenous mouse p53 and mouse PKR in mouse cells, mediated by MAV-1 E4orf6 and E1B 55K during infection. Another hAd E4 protein, E4orf3, causes proteasomal degradation of transcriptional intermediary factor 1γ (78) and general transcription factor II-I (79) in a manner independent of hAd E4orf6 and E1B 55K. E4orf3 has SUMO E3 ligase and E4 elongase activity and induces sumoylation of general transcription factor II-I, leading to its proteasome-dependent degradation (79). MAV-1 E4orf3 may similarly have sumoylation activity that results ultimately in proteasome-dependent PKR degradation. Another possibility of a viral protein involved in PKR degradation is the protease encoded by MAV-1. The hAd protease is encapsidated in virions and proteolytically processes viral proteins IIIa, VI, VII, VIII, mu, and TP (80–83). However, we think it is unlikely that the MAV-1 protease degrades PKR, because we showed that UV-inactivated virus was unable to degrade PKR. We assume that UV treatment would not destroy the MAV-1 protease activity, just as HSV-1 VP16 activity is not altered by UV inactivation of HSV-1 (84), but we have not tested this directly.

In summary, we demonstrated that PKR has an antiviral role during MAV-1 infection *in vitro*, because when PKR is mutated, viral replication in MEFs is significantly higher than that seen in wild-type MEFs. Analysis of global PKR steady-state protein levels during infection showed complete PKR depletion by 72 hpi in multiple cell types, including immortalized and primary cells, with even faster kinetics in some. PKR transcription and translation were not decreased by MAV-1 infection, whereas proteasomal inhibition prevented PKR degradation. Taken together, these data suggest that MAV-1 causes PKR to be proteasomally degraded at a posttranslational level. This work provides new insight into possible mechanisms of adenovirus inhibition of PKR by DNA viruses. PKR degradation may be induced by other adenoviruses that do not produce VA RNA, which includes all animal adenoviruses except primate adenoviruses and one type of fowl adenovirus (85).

## MATERIALS AND METHODS

### Cells, virus, and infections.

CMT93 cells (CCL-223) and C57BL/6 MEFs (SCRC-1008) were obtained from the American Type Culture Collection and passaged in Dulbecco’s modified Eagle medium (DMEM) containing 5% and 10% heat-inactivated fetal bovine serum (FBS), respectively, before use. Primary peritoneal macrophages were obtained from 6-to-10-week-old C57BL/6 mice purchased from Jackson Laboratory (catalog no. 000664) as described previously (86). Briefly, 6-to-10-week-old C57BL/6 mice were injected intraperitoneally with 1.2 ml 3% thioglycolate and euthanized 3 to 5 days later. The abdominal skin was carefully removed, exposing the peritoneum, which was then injected with 5 ml of sterile phosphate-buffered saline (PBS). The abdomen was massaged gently, and then the PBS containing the peritoneal macrophages was carefully withdrawn. The macrophages were centrifuged at 100 × *g* for 4 min, and red blood cells were lysed in lysis buffer (0.15 M ammonium chloride, 1 mM potassium bicarbonate, 0.1 mM EDTA disodium salt) for 2 min at room temperature, centrifuged at 100 × *g* for 4 min, washed twice in PBS, resuspended in DMEM–5% heat-inactivated FBS, and plated in 6-well plates. WT and PKR^−/−^ MEFs (termed PKR WT MEFs and N-PKR^−/−^ MEFs, respectively, throughout this paper) were obtained from Robert Silverman, Cleveland Clinic (87), and were passaged in DMEM containing 10% heat-inactivated FBS before use. PKR^−/−^ MEFs stably transfected with empty vector (termed C-PKR^−/−^ MEFs throughout this paper) were obtained from Gokhan Hotamisligil, Harvard University (88), and were passaged in DMEM containing 10% heat-inactivated FBS before use. WT (SV40 MEFs) and K271R PKR mutant (K271R SV40 MEFs) MEFs were obtained from Anthony Sadler, Hudson Institute of Medical Research (54), and were passaged in DMEM containing 10% heat-inactivated FBS before use.

Wild-type mouse adenovirus type 1 (MAV-1) stock was prepared, and titers were determined on mouse NIH 3T6 fibroblasts as described previously (89). WT MAV-1 was subjected to UV inactivation by UV treatment of 200 µl of virus for 10 min at 800 mJ/cm^2^. UV inactivation was confirmed by qPCR and plaque assay.

For infection assays, medium was removed from cells and adsorption procedures were performed with 0.4 ml of inocula in 6-well plates with 35-mm-diameter wells (unless otherwise noted) for 1 h at 37°C at the indicated MOIs (PFU/cell). After 60 min, 2 ml of DMEM–5% FBS was added without removal of inocula; that time point was designated 0 hpi. For araC experiments, 20 µg/ml araC (Sigma C1768) was added at 0 hpi and replenished every 12 to 16 h.

### Immunoblotting.

At room temperature, cells were washed once with PBS, and Pierce radioimmunoprecipitation assay (RIPA) lysis buffer (Thermo Scientific catalog no. 89900) with 1× protease inhibitors (protease inhibitor cocktail kit; Thermo Scientific catalog no. 78410) was added to the plate. The cells were allowed to lyse at room temperature for 10 min before being harvested and centrifuged at 4°C at 14,000 × *g* for 10 min to remove debris. Equivalent amounts of protein, determined by a bicinchoninic acid (BCA) assay (Pierce BCA protein assay kit; Thermo Scientific catalog no. 23227), were subjected to acetone precipitatation by incubation with a 4× volume of ice-cold acetone overnight at −20°C. Precipitated proteins were pelleted at 4°C at 13,000 × *g* for 10 min, and the pellets were dried for 30 min at room temperature. Pellets were resuspended in a mixture of 10 µl Pierce RIPA lysis buffer (Thermo Scientific catalog no. 89900), 3.25 µl NuPAGE 4× lithium dodecyl sulfate (LDS) sample buffer (Invitrogen catalog no. NP0007), and 1.25 µl 1 M dithiothreitol (DTT). Samples were incubated at 37°C for 10 min and then loaded into a well of an 8% acrylamide gel (8.3 cm wide by 7.3 cm high by 0.1 cm thick) with a 2.5% stacking gel, electrophoresed for 30 min at 50 V and 85 min at 150 V, and then transferred to a polyvinylidene difluoride (PVDF) membrane (Bio-Rad catalog no. 1620177) for 1 h at 100 V at 4°C. Blots were blocked in 5% bovine serum albumin (BSA; Sigma catalog no. A7906)–Tris-buffered saline (Bio-Rad catalog no. 1706435)–0.1% Tween 20 (Sigma catalog no. P1379). Blots were probed with primary antibodies to detect mouse PKR (Santa Cruz D-20 sc-708 [1:2,000] or B-10 sc-6282 [1:200]), mouse actin (Santa Cruz sc-1616-R [1:1,000]), MAV-1 E1A (AKO-7-147 [1:1,000]; described previously [67]), or MAV-1 late viral proteins (AKO 1-103 [1:1,000]; described previously [90, 91]). The secondary antibodies used were IRDye 800CW anti-rabbit (Li-Cor 925-32213 [1:15,000]) or IgG peroxidase-conjugated anti-mouse (Jackson Immuno 515-035-062 [1:20,000]) antibody. Blots were visualized by the use of Li-Cor Odyssey imaging (Li-Cor Biosciences) or enhanced chemiluminescent substrates (Pierce ECL Western blotting substrate; catalog no. 32106) and X-ray film (Dot Scientific catalog no. BDB810). Densitometric quantification was performed using .tif files and ImageJ software from NIH (92).

To attempt to demonstrate PKR ubiquitination status during MAV-1 infection, C57BL/6 MEFs were transfected with green fluorescent protein (GFP) (Addgene catalog no. 11928)-tagged or hemagglutinin (HA) epitope (Addgene catalog no. 18712)-tagged ubiquitin plasmids 24 h before infection. We used Polyplus jetPRIME transfection reagent (Polyplus catalog no. 114-15) with 10 µg plasmid DNA and 30 µl jetPRIME reagent per 10-cm-diameter plate. At 12 hpi (36 h posttransfection), we treated mock-infected and infected C57BL/6 MEFs with 10 µM MG132 (Sigma M7449) for 6 h before collecting lysates at 18 hpi in HCN buffer (50 mM HEPES, 150 mM NaCl, 2 mM CaCl_2_, 1% Triton X-100 [Sigma T9284]), 1× protease inhibitors [protease inhibitor cocktail kit; Thermo Scientific catalog no. 78410], 5 mM N-ethylmaleimide). The lysates were split into two aliquots, and 3 µg PKR (Santa Cruz D-20 sc-708; discontinued) or 3 µg isotype rabbit polyclonal antibody (Jackson Immuno catalog no. 011-000-002) was added to lysates. After rocking samples overnight at 4°C, 20 µl of a protein A agarose suspension (Calbiochem/Millipore catalog no. IP02-1.5ML) was added to each sample and the samples were rocked at 4°C for 2 h. After incubation, the agarose was washed 3 times with 1 ml HCN buffer, resuspended in 40 µl 2× Laemmli buffer (Bio-Rad catalog no. 161-0737)–5% 2-mercaptoethanol (Sigma M6250), and boiled for 10 min. The lysate supernatants remaining after the initial PKR immunoprecipitation were then immunoprecipitated again using the same procedure but with 4 µg anti-p53 mouse monoclonal antibody (DO-1; Santa Cruz sc-126) or 4 µg isotype mouse monoclonal antibody (ThermoFisher Scientific catalog no. 02-6200). Immunoprecipitated proteins were subjected to immunoblotting for GFP or HA epitope-tagged ubiquitin with antibodies for GFP (Roche catalog no. 11814460001) (1:3,000) or HA (Abcam catalog no. ab9110) (1:4,000). No ubiquitin signal was detected from the PKR immunoprecipitations, though the positive control, p53, showed ubiquitin signal with both epitope-tagged ubiquitins (Fig. S5 in the supplemental material). Blots were also probed for PKR (PKR B-10 sc-6282) (1:200), p53 (anti-p53 sc-98) (1:200), and IRDye 800CW anti-mouse (Li-Cor 925-32212) (1:15,000) to confirm that the immunoprecipitations had been successful, and signals for both proteins were detected (Fig. S5).

### Viral DNA yield analysis by qPCR.

Cells were washed twice with room temperature PBS and harvested by scraping into PBS, centrifuging at 100 × *g* for 4 min at 4°C, and resuspending in PBS. Total cellular DNA was purified using an Invitrogen PureLink DNA purification kit (Thermo Scientific catalog no. K1820-02) and quantitated using a NanoDrop spectrophotometer. A 10-ng volume of total cellular DNA was analyzed by qPCR using custom primers specific to MAV-1 E1A (mE1Agenomic Fwd [5′ GCA CTC CAT GGC AGG ATT CT 3′] and mE1Agenomic Rev [5′ GGT CGA AGC AGA CGG TTC TTC 3′]), and the results were normalized to GAPDH (glyceraldehyde-3-phosphate dehydrogenase), which was analyzed using a GAPDH-specific primer/probe set (Thermo Fisher Scientific Mm99999915_g1; catalog no. 4331182).

### mRNA analysis by qPCR.

Cells were harvested by scraping into media, centrifuging at 100 × *g* for 4 min at 4°C, and washing the cell pellet three times with ice-cold PBS. RNA was purified using a Qiagen RNeasy minikit (Qiagen catalog no. 74134) and stored at −80˚C. A 125-ng volume of RNA per sample was used to make cDNA using a High-Capacity cDNA reverse transcription (RT) kit (Applied Biosystems catalog no. 4368814), and 2 µl of the cDNA was analyzed by qPCR using a primer/probe set specific to mouse PKR sequence (Thermo Fisher Mm01235643_m1; catalog no. 4331182). The results were normalized to GAPDH, which was analyzed using a GAPDH-specific primer/probe set (Thermo Fisher Scientific Mm99999915_g1; catalog no. 4331182). Arbitrary units were calculated as follows: Mean threshold cycle (*C_T_*) PKR − mean *C_T_* GAPDH = Δ*C_T_* for sample (arbitrary unit = 2^−Δ^*^CT^*).

### Proteasome inhibition.

C57BL/6 MEFs were infected at an MOI of 10, and DMSO, 1 µM MG132 (Sigma M7449), or 1 µM bortezomib (Selleckchem catalog no. S1013) was added to the media after a 1 h adsorption. At 24 hpi, cells were washed once with room temperature PBS, and Pierce RIPA lysis buffer (Thermo Scientific catalog no. 89900) with 1× protease inhibitors (protease inhibitor cocktail kit; Thermo Scientific catalog no. 78410) was added to the plate. The cells were allowed to lyse at room temperature for 10 min before being harvested and centrifuged at 4°C at 14,000 × *g* for 10 min to remove debris.

### Lysosome inhibition and DQ BSA assay.

CMT93 cells were infected at an MOI of 10. After 1 h of adsorption, 10 µl water, 10 mM (final concentration) NH_4_Cl (Baker Chemical Company catalog no. 0660-1), or 60 µM (final concentration) chloroquine (Sigma C6628) was added to the media. At 24 hpi, at room temperature, cells were washed once with PBS, and Pierce RIPA lysis buffer (Thermo Scientific catalog no. 89900) with 1× protease inhibitors (protease inhibitor cocktail kit; Thermo Scientific catalog no. 78410) was added to the plate. The cells were allowed to lyse at room temperature for 10 min before being harvested and centrifuged at 4°C at 14,000 × *g* for 10 min to remove debris.

The DQ BSA assay was performed as described previously (59). Briefly, C57BL/6 MEFs and CMT93 cells were plated at 1.5 × 10^5^ cells/plate and 3 × 10^5^ cells/plate, respectively, in MatTek glass-bottom microwell dishes (part no. P35G-1.5-14C) with 2 ml of DMEM–10% and DMEM–5% FBS, respectively. The next day, the cell medium was treated with 10 µl water, 10 mM (final concentration) NH_4_Cl, or 60 µM (final concentration) chloroquine (Sigma C6628). Four hours after addition of inhibitors, DQ red BSA (Invitrogen catalog no. D12051) was added to the media to reach a final concentration of 5 µg/ml in 2 ml DMEM plus 10% and 5% FBS, respectively. At 24 h posttreatment, the cells were imaged on a Nikon TE300 inverted microscope equipped with a mercury arc lamp; a Plan-Apochromat 60×, 1.4-numerical-aperture (NA) objective; a cooled digital charge-coupled-device (CCD) camera (Quantix Photometrics, Tucson, AZ); and a temperature-controlled stage (set at 37°C). To image the DQ-BSA, we used an excitation filter centered at 572 nm and an emission filter centered at 635 nm. The exposure times were the same for all images.

### Ribosome pelleting.

Ribosomes were pelleted as described previously (93). Briefly, C57BL/6 MEFs were plated on 10-cm-diameter plates at 3 × 10^5^ cells per plate. The next day, the cells (∼90% confluent) were infected with MAV-1 at an MOI of 5. C57BL/6 MEF lysates were collected at 48 hpi by scraping the cells in ice-cold PBS containing 100 µg/ml cycloheximide (Sigma C7698), pelleting, and resuspending in lysis buffer, which contained 10 mM HEPES (pH 7.5), 100 mM KCl, 5 mM MgCl_2_, 4 mM DTT, 0.5% NP-40, 100 µg/ml cycloheximide, 20 U/ml RNasin (Promega catalog no. N2511), 10% sucrose, and 1× protease inhibitors (protease inhibitor cocktail kit; Thermo Scientific catalog no. 78410). Cells were lysed by passage through a chilled 26-gauge needle five times and were cleared by centrifugation for 10 min at 21,000 × *g* at 4°C. A 400-µl volume of cleared lysate (optical density at 260 nm [OD_260_] of 10) was layered onto 25% sucrose and centrifuged at 29,500 rpm in an SW41 rotor (average relative centrifugal force [rcf], 107,458) for 4 h at 4°C. After pelleting, the supernatant was removed by the use of a micropipette, and 350 µl of Buffer RLT Plus (from Qiagen RNeasy minikit) (4°C) was added to the pellet for collection of the RNA. The RNA was purified immediately using an Qiagen RNeasy minikit (Qiagen catalog no. 74134) and stored at −80˚C until analysis.

### Polyribosome gradients.

C57BL/6 MEFs were plated on 10-cm-diameter plates (2 × 10^6^ cells per plate). The next day, the cells were infected with MAV-1 at an MOI of 2. Following a standard protocol (94), 5 min prior to collection, cycloheximide was added at a final concentration of 100 µg/ml and the reaction mixture was incubated at 37°C. Cells were collected at 24 hpi by scraping in ice-cold PBS containing 100 µg/ml cycloheximide, pelleting, and resuspending in 500 µl lysis buffer (20 mM Tris-Cl, 150 mM NaCl, 15 mM MgCl_2_, 8% glycerol, 20 IU/ml SUPERase•In [Thermo Fisher Scientific catalog no. AM2696], 80 IU/ml murine RNase inhibitor [New England BioLabs catalog no. M0314S], 0.1 mg/ml heparin [Sigma H3393-50], 0.1 mg/ml cycloheximide, 1 mM DTT, 1× protease inhibitor [protease inhibitor cocktail kit; Thermo Scientific catalog no. 78410], 20 IU/ml Turbo DNase [Thermo Fisher Scientific catalog no. AM2238], 1% Triton X-100 [Sigma T9284]). Cells were lysed by passage through a chilled 26-gauge needle 10 times, vortex mixing for 30 s, and then incubating on ice for 5 min. Lysates were cleared by centrifugation for 5 min at 14,000 × *g* at 4°C. A 500-µl volume of cleared lysate (OD_260_ of 10) was layered onto a 10%-to-50% sucrose gradient and centrifuged at 35,000 rpm in an SW41 rotor (151,000 rcf) for 3 h at 4°C. After centrifugation, gradients were pumped out of the top with a Brandel BR-188 density gradient fractionation system with a continuous reading of the OD_254_. From 24 to 34 fractions (350 to 500 µl) were collected. RNA was purified from selected fractions immediately using a Qiagen RNeasy minikit (Qiagen catalog no. 74134) and stored at −80˚C until analysis by RT-qPCR.

### Statistical analyses.

Data were analyzed with GraphPad Prism 7.0 software. For qPCR and densitometry analyses, the data were analyzed by individual Mann-Whitney tests. A *P* value of *<*0.5 was considered significant.
