# Supplementary material for: Enhanced Replication of Mouse Adenovirus Type 1 following Virus-Induced Degradation of Protein Kinase R (PKR)
Source: mBio. 2019 Apr 23;10(2):e00668-19. doi: 10.1128/mBio.00668-19 (PMC6479006; doi:10.1128/mBio.00668-19)
Supplement: FIG S1 [file mBio.00668-19-sf001.pdf]

Supplemental Figure 1

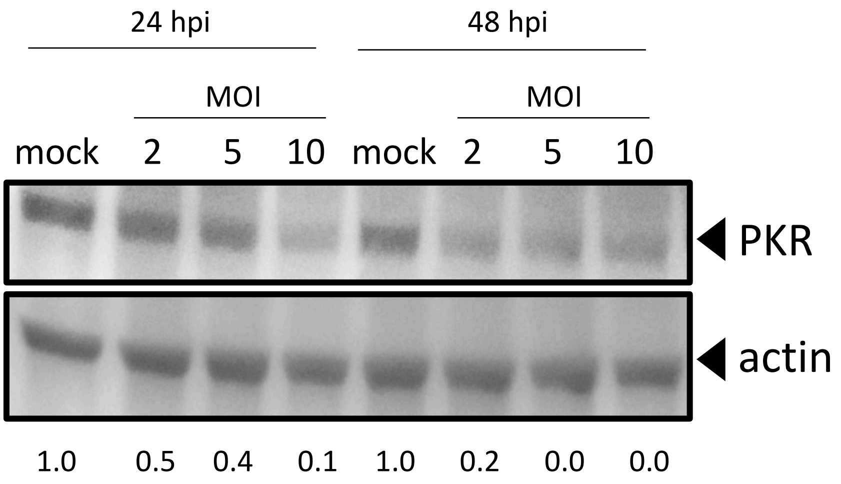

Supplemental Figure 1. PKR is depleted at MOIs of 2, 5, and 10. C57BL/6 MEFs were infected with MAV-1 at an MOI of 2, 5, or 10 (2, 5, 10) or mock infected (mock). Cell lysates were analyzed by immunoblot with antibodies for PKR (B-10) and actin. Densitometry quantitation using ImageJ is listed below each lane; for each time point, the infected samples were normalized to the mock. The PKR blot image was uniformly adjusted to a brightness of 150 in Adobe Photoshop.
