# Supplementary material for: Enhanced Replication of Mouse Adenovirus Type 1 following Virus-Induced Degradation of Protein Kinase R (PKR)
Source: mBio. 2019 Apr 23;10(2):e00668-19. doi: 10.1128/mBio.00668-19 (PMC6479006; doi:10.1128/mBio.00668-19)
Supplement: FIG S2 [file mBio.00668-19-sf002.pdf]

Supplemental Figure 2

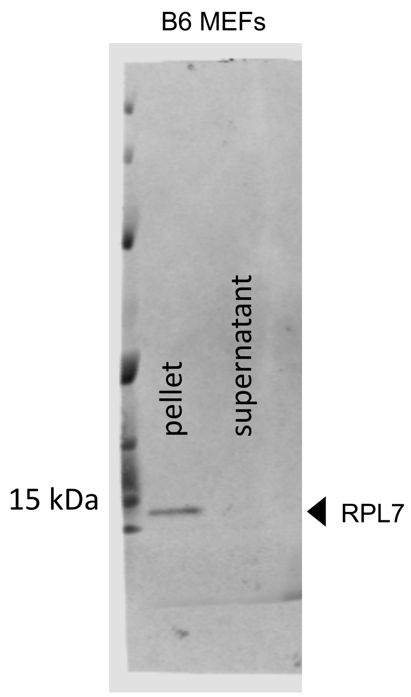

Supplemental Figure 2. Ribosome pelleting. To confirm that most ribosomes ended up in the pellet after centrifugation through sucrose (Fig. 4A), a sample of the pellet and the corresponding supernatant were analyzed by immunoblot with antibodies for RPL7 (ribosomal protein L7, Abcam, 1:2000, ab72550).
