# Supplementary material for: Enhanced Replication of Mouse Adenovirus Type 1 following Virus-Induced Degradation of Protein Kinase R (PKR)
Source: mBio. 2019 Apr 23;10(2):e00668-19. doi: 10.1128/mBio.00668-19 (PMC6479006; doi:10.1128/mBio.00668-19)
Supplement: FIG S3 [file mBio.00668-19-sf003.pdf]

Supplemental Figure 3

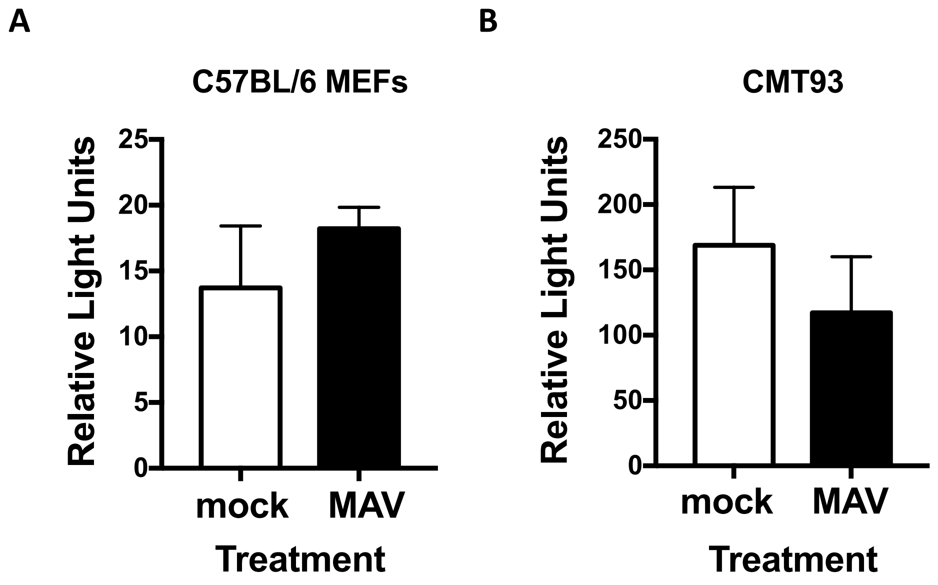

Supplemental Figure 3. PKR mRNA 5' UTR does not result in altered reporter protein levels upon MAV-1 infection. (A) C57BL/6 MEFs or (B) CMT93 cells were co-transfected with pmPKR5UTRfullNL or AUG-NL-3xFLAG and pGL4.13 using jetPRIME reagents (Polyplus #114-15) using the standard Polyplus protocol, with 200 ng total of plasmid and 300 μL of jetPRIME reagent per 35 mm well. At 24 hours after transfection, the cells were infected with MAV-1 at an MOI of 10. At 24 hpi, cells were lysed in 70 μL/well Glo Lysis Buffer (Promega Corp.). After lysing, 25 μL of each lysed sample and 25 μL of OneGlo or NanoGlo (Promega Corp.) was added to two wells in a black 96-well plate (Fisher Scientific #07-000-634). After 5 minutes, the plate was read on a Promega GloMax luminometer. Relative light units from the pmPKR5UTRfullNL plasmid were normalized to the firefly luciferase and positive control plasmids. Graphs are representative of 7-9 biological replicates per treatment group.
