# Supplementary material for: Enhanced Replication of Mouse Adenovirus Type 1 following Virus-Induced Degradation of Protein Kinase R (PKR)
Source: mBio. 2019 Apr 23;10(2):e00668-19. doi: 10.1128/mBio.00668-19 (PMC6479006; doi:10.1128/mBio.00668-19)
Supplement: FIG S4 [file mBio.00668-19-sf004.pdf]

Supplemental Figure 4

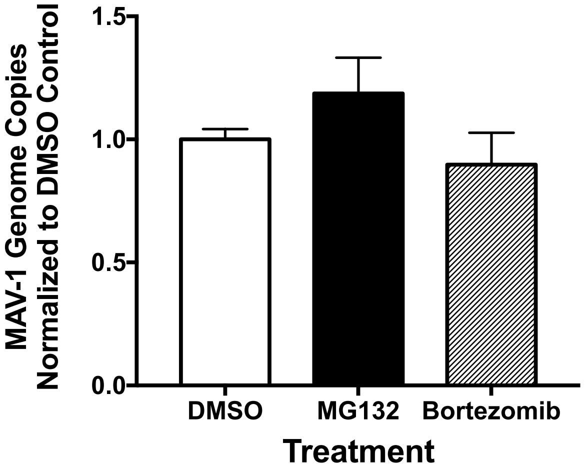

Supplemental Figure 4. MG132 and bortezomib treatment do not affect MAV-1 replication at 24 hpi. C57BL/6 MEFs were infected with MAV-1 at an MOI of 10 and treated with DMSO (vehicle for inhibitors), 1 μM MG132 or bortezomib, and collected at 24 hpi. DNA was purified from cell pellets and analyzed for MAV-1 genome copies by qPCR. Graph is representative of five biological replicates per treatment group. Error bars are standard error of the mean (SEM). $*P \leq 0.05$
