# Supplementary material for: Enhanced Replication of Mouse Adenovirus Type 1 following Virus-Induced Degradation of Protein Kinase R (PKR)
Source: mBio. 2019 Apr 23;10(2):e00668-19. doi: 10.1128/mBio.00668-19 (PMC6479006; doi:10.1128/mBio.00668-19)
Supplement: FIG S5 [file mBio.00668-19-sf005.pdf]

Supplemental Figure 5

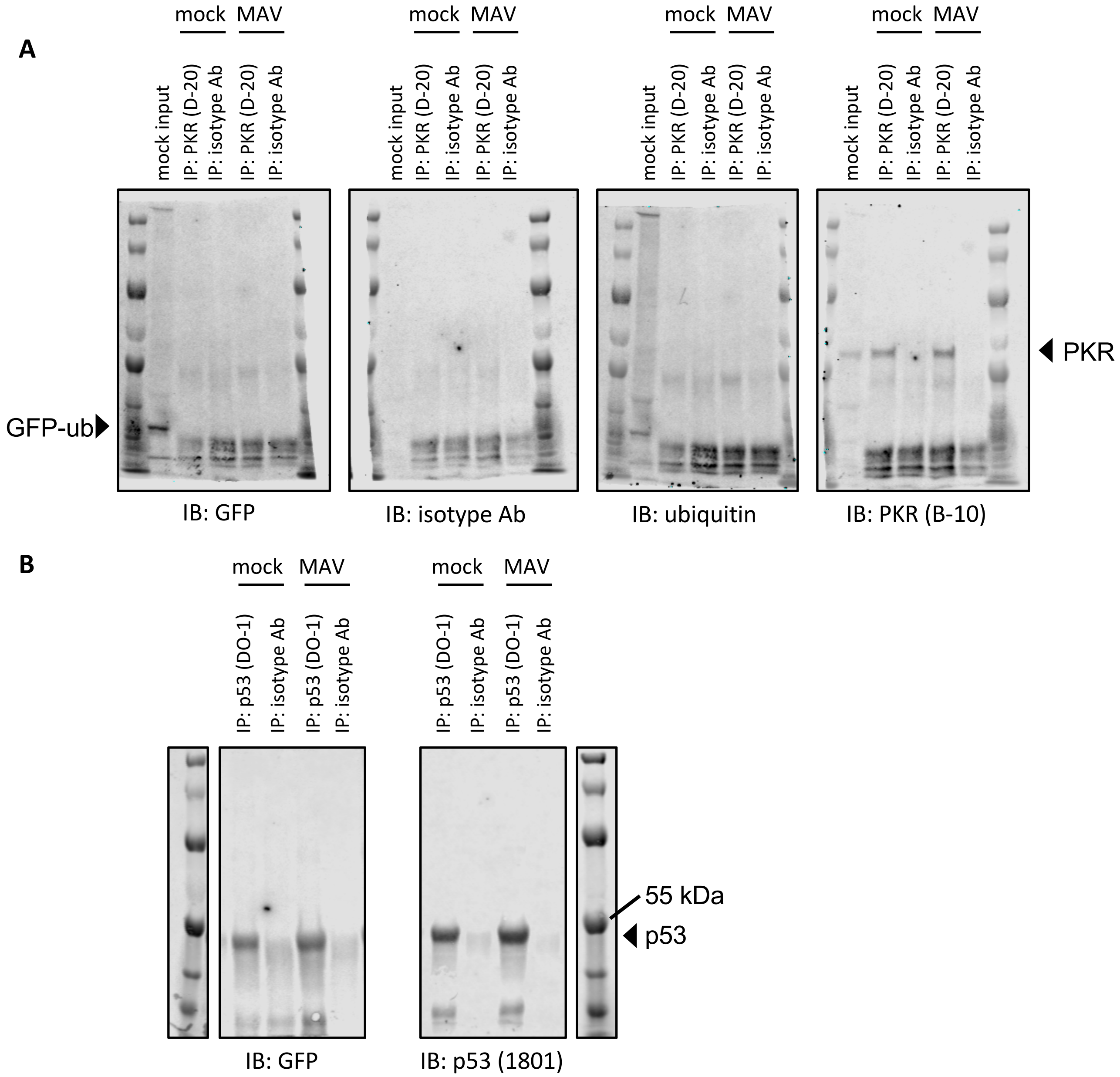

Supplemental Figure 5. PKR is not detectably ubiquitinated during MAV-1 infection. CMT93 cells were transfected with a GFP-ubiquitin plasmid using standard Polyplus transfection protocols. At 24 hpt, the cells were infected with MAV-1 (MAV) at an MOI of 5 or mock infected (mock) and treated with 10 μM MG132 at 6 hpi. Cell lysates were collected at 12 hpi and immunoprecipitated with (A) PKR (D-20) or an isotype control antibody or (B) p53 (DO-1) or an isotype control antibody. Immunoprecipitated samples were analyzed by immunoblot with GFP, ubiquitin, isotype, PKR (B-10), or p53 (1801) antibodies. Input lane (mock input) contains 0.008 volume of mock infected lysate (relative to volume in immunoprecipitations). For (B) GFP and p53 were probed on separate duplicate blots.
