# Supplementary material for: Enhanced Replication of Mouse Adenovirus Type 1 following Virus-Induced Degradation of Protein Kinase R (PKR)
Source: mBio. 2019 Apr 23;10(2):e00668-19. doi: 10.1128/mBio.00668-19 (PMC6479006; doi:10.1128/mBio.00668-19)
Supplement: FIG S6 [file mBio.00668-19-sf006.pdf]

Supplemental Figure 6

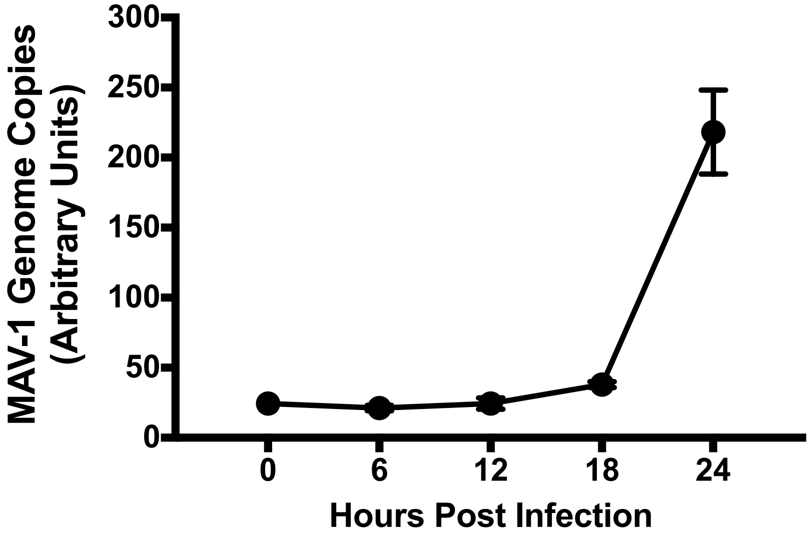


**Supplemental Figure 6.** Viral DNA replication can be detected at 24 hpi by qPCR. CMT93 cells were infected with MAV-1 (MAV) at an MOI of 10 and collected every 6 hours for 24 hours. DNA was purified from cell pellets and analyzed for MAV-1 genome copies by qPCR. Graph is representative of four to five biological replicates per treatment group. Error bars are standard error of the mean (SEM).
