# Supplementary material for: Enhanced Replication of Mouse Adenovirus Type 1 following Virus-Induced Degradation of Protein Kinase R (PKR)
Source: mBio. 2019 Apr 23;10(2):e00668-19. doi: 10.1128/mBio.00668-19 (PMC6479006; doi:10.1128/mBio.00668-19)
Supplement: FIG S7 [file mBio.00668-19-sf007.pdf]

Supplemental Figure 7

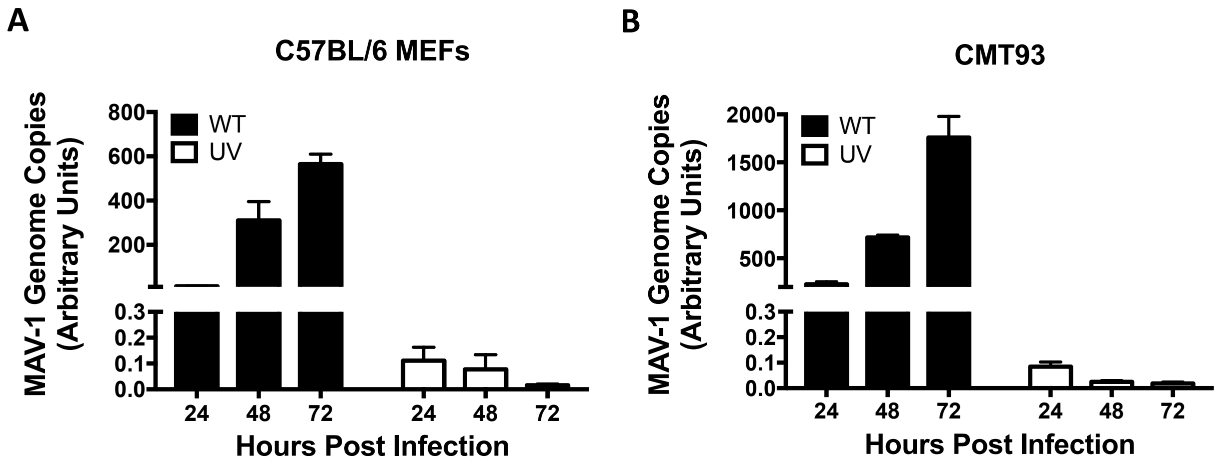

Supplemental Figure 7. UV-inactivated virus does not replicate viral DNA. (A) C57BL/6 MEFs or (B) CMT93 cells were infected with WT MAV-1 (WT) or UV-inactivated MAV-1 (UV) at an MOI of 10 and collected at indicated times. DNA was purified from cell pellets and analyzed for MAV-1 genome copies by qPCR. Graphs are representative of three to four biological replicates per treatment group. Error bars are standard error of the mean (SEM).
