# Supplementary material for: Enhanced Replication of Mouse Adenovirus Type 1 following Virus-Induced Degradation of Protein Kinase R (PKR)
Source: mBio. 2019 Apr 23;10(2):e00668-19. doi: 10.1128/mBio.00668-19 (PMC6479006; doi:10.1128/mBio.00668-19)
Supplement: FIG S8 [file mBio.00668-19-sf008.pdf]

Supplemental Figure 8

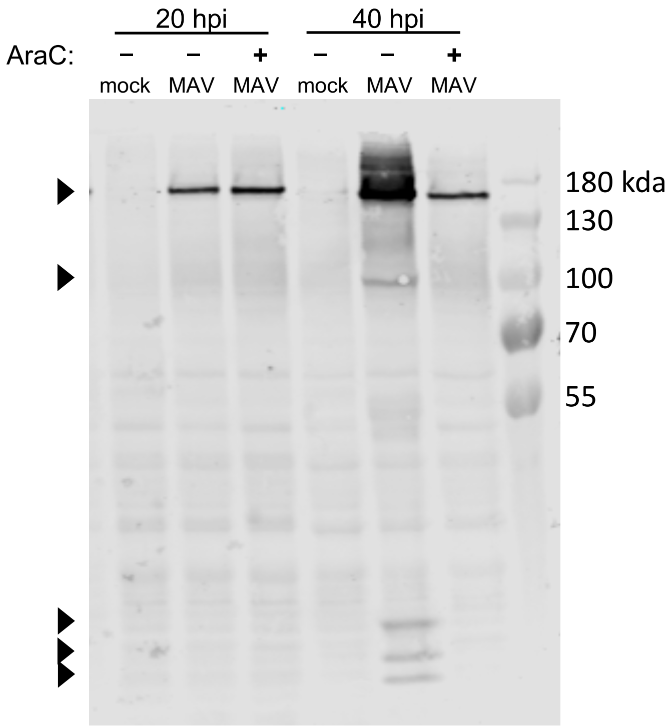

Supplemental Figure 8. AraC treatment inhibited late protein expression. CMT93 cells were infected with WT MAV-1 (MAV) at an MOI of 10 or mock infected (mock). Infected cells were also treated (+) or not (-) with 20 μg/mL cytosine arabinasine (araC), an inhibitor of DNA synthesis. Cell lysates were analyzed with antibodies for late virion proteins (AKO1-103, 1:1000). Arrowheads indicate late viral proteins.
